# The effect of phylogeographic history on species boundaries: a comparative framework in *Hyla* tree frogs

**DOI:** 10.1038/s41598-020-62382-4

**Published:** 2020-03-26

**Authors:** Christophe Dufresnes, Matthieu Berroneau, Sylvain Dubey, Spartak N. Litvinchuk, Nicolas Perrin

**Affiliations:** 1 LASER, College of Biology and the Environment, Nanjing Forestry University, Nanjing, China; 2 Department of Ecology & Evolution, University of Lausanne, Lausanne, Switzerland; 3 Cistude Nature, Le Haillan, France; 4 Agrosustain SA, Nyon, Switzerland; 5 Hintermann & Weber SA, Montreux, Switzerland; 6 Institute of Cytology, Russian Academy of Sciences, Saint Petersburg, Russia; 7 Dagestan State University, Makhachkala, Russia

**Keywords:** Herpetology, Speciation, Taxonomy, Phylogenetics, Population genetics, Biogeography, Molecular ecology

## Abstract

Because it is indicative of reproductive isolation, the amount of genetic introgression across secondary contact zones is increasingly considered in species delimitation. However, patterns of admixture at range margins can be skewed by the regional dynamics of hybrid zones. In this context, we posit an important role for phylogeographic history: hybrid zones located within glacial refugia (putatively formed during the Late-Pleistocene) should be better defined than those located in post-glacial or introduced ranges (putatively formed during the Holocene and the Anthropocene). We test this hypothesis in a speciation continuum of tree frogs from the Western Palearctic (*Hyla*), featuring ten identified contacts between species spanning Plio-Pleistocene to Miocene divergences. We review the rich phylogeographic literature of this group and examine the overlooked transition between *H. arborea* and *H. molleri* in Western France using a multilocus dataset. Our comparative analysis supports a trend that contacts zones resulting from post-glacial expansions and human translocations feature more extensive introgression than those established within refugial areas. Integrating the biogeographic history of incipient species, i.e. their age since first contact together with their genetic divergence, thus appears timely to draw sound evolutionary and taxonomic inferences from patterns of introgression across hybrid zones.

## Introduction

How and when the continuous process of speciation leads to discrete entities is a central question in evolutionary biology and systematics^1^. Reproductive isolation evolves as diverging lineages accumulate genetic changes, ultimately reducing the probability of mating (pre-zygotic barriers) and the fitness of interspecific hybrids (post-zygotic barriers)^2^. Hybridizing taxa thus lay at the boundaries of the biological species concept^3^ and offer opportunities to understand how much divergence is needed to maintain incipient species apart, despite occasional gene flow^4^.

Since it reflects the balance between dispersal and selection against hybrids, the amount of admixture at phylogeographic transitions serves as an ad hoc measure of reproductive isolation under natural conditions. Although scarce, comparative hybrid zone analyses support a gradual buildup of reproductive isolation with the time diverging lineages have spent in allopatry, where a remarkably short temporal window separates widely admixing and genetically isolated taxa^5–7^. Characterizing this “grey zone of speciation” is attractive for species delimitation^7^. Hence, assessing the amount of admixture at phylogeographic transitions is increasingly viewed as a key step in integrative taxonomy, notably for controversial species^8^.

However, patterns of introgression at hybrid zones can also be influenced by an array of extrinsic factors that confound with reproductive isolation, e.g. local barriers to gene flow due to landscape features, demographic events of extinction and recolonization, or changes in ecological conditions shifting species transitions^6,9–12^. We posit that the time since contact should also play a major role, in the light of the biogeographic history of species. In the Northern hemisphere, the Quaternary climatic oscillations frequently remodeled lineage distributions and set up the contact zones observed nowadays^13^. Species transitions located in glacial refugia may have existed since or even prior to the last glaciation, and may thus be as old as several hundred thousand of years. In contrast, contact zones formed by post-glacial recolonization post-date the Last Glacial Maximum (LGM, ~21,000 years ago), and are no older than a few thousands of years. Post-glacial expansions should lead to wide, mosaic parapatric distributions as species colonized new, empty ranges, across the climatically instable northern regions, giving rise to multiple local contacts that may not have reached their ecological or genetic equilibrium yet (e.g.^12^). As a result, these transitions would exhibit admixture over large distances, even if the interacting species are partially reproductively isolated. Reciprocally, contact zones that have persisted in glacial refugia had more time to purge hybrid genotypes and should feature more stable transitions. These processes could also affect opportunities for reinforcement, i.e. the evolution of assortative mating as a response to selection against unfit or maladapted hybrids^14,15^. Species could start establishing pre-mating barriers earlier in refugial than in post-glacial ranges, where they are thus expected to experience less gene flow.

Given these contrasting outcomes, did the biogeographic history of secondary contacts significantly affect their amount of admixture, in turn blurring species delimitation assessments? This question is fundamental for both speciation and systematic biology, but because the answer requires replicate transitions between the same species pairs, or at least between pairs of similar divergence, it has never been formally tested. Several unrelated studies yet support remarkable latitudinal differences in gene flow. The rate of hybridization between the collared and pied flycatchers (*Ficedula*) is higher in the Baltic Isles than in Central Europe^16^, where both species have been in sympatry for a longer time and evolved character displacement on male plumage color via reinforcement^17^. Time since contact was also invoked to explain differences in hybrid zone structures among *Quercus* oaks^18^. In house spiders (*Tegenaria*), two sister species co-exist in England and widely hybridize in northern, but not in southern parts of the country^19^. These scattered observations match our expectations of wider hybrid zones in recently colonized ranges, for the same species pair.

The Western-Palearctic radiation of tree frogs (*Hyla*) provides a unique framework to compare phylogeographic transitions in respect to their late-Quaternary history. The phylogeny and phylogeography of this group have been extensively characterized over the last decade, for all species individually, but also at range margins. W-Palearctic tree frogs diversified from the Miocene (>10 Ma) to the Plio-Pleistocene (3–4 Ma)^20^ and now include ten species forming replicate transitions across refugial, post-glacial, or even introduced ranges. Here we revisit their patterns of admixture in the light of the history of contacts. We first detail the overlooked hybrid zone between *H. arborea* and *H. molleri* preliminary located in W-France^21–23^, using a population genetic approach. This pair is of high comparative interest since it diverged almost simultaneously (5–6 Ma) with several other species pairs (*H. arborea*/*orientalis*, *H. arborea*/*perrini*, *H. savignyi*/*felixarabica*). Second, we assess how admixture across phylogeographic transitions varies depending on species divergence and relative time since first contact, by combining independently published data from ten contact zones.

## Methods

### *Population genetics of the* Hyla arborea*/*molleri *hybrid zone*

A total of 251 adult tree frogs (*n* = 231 *H. arborea/molleri*, as well as 20 syntopic *H. meridionalis*) were captured from 25 localities (loc. 4–29) throughout Western France (File S1). DNA was collected with non-invasive buccal swabs, stored dry at −20 °C, and extracted with the BioSprint robotic workstation (Qiagen). The experiments conducted in this research (DNA sampling) were performed in accordance with the regional authorities (Prefectures of Ille-et-Vilaine, Charente and Gironde) under collecting permits, and relevant guidelines were followed. No individuals were harmed or killed, and all were released at their place of capture.

Twenty-six microsatellite loci cross-amplifying in *H. arborea* and *H. molleri* were genotyped in all samples: *Ha*-T3, *Ha*-T52, *Ha*-M2, WHA5–22, *Ha*-T45, *Ha*-T11, *Ha*-T49, *Ha*-T51, *Ha*-T64, *Ha*-T41, *Ha*-T69, *Ha*-T54, *Ha*-T55, *Ha*-T50, *Ha*-T58, *Ha*-T53, *Ha*-T56, *Ha*-T66, *Ha*-T60, *Ha*-T61, *Ha*-T68, *Ha*-T63, *Ha*-T67, WHA1–25, WHA1–67, *Ha*-A11^24,25^ (see methods therein). Eight loci did not amplify in *H. meridionalis*, in line with the older divergence time. In addition, we re-analyzed microsatellite reads from reference populations of *H. arborea* (from Switzerland, the Netherlands, and France, loc. 1–3, *n* = 80), *H. molleri* (from Spain, loc. 30, *n* = 16) and *H. meridionalis* (from southern France, loc. 31, *n* = 16) from our previous work^24–26^. The new and published genotypes were generated by the same genetic analyzer (an ABI3130) and (re)scored together in GenMapper 4.0 with a single set of bins and panels, to ensure the correspondence of alleles.

The total microsatellite dataset (Western France + reference populations) included 363 individuals. We first performed Bayesian clustering with STRUCTURE^27^ by running 10 chains of 100’000 iterations, after a burnin of 10’000, for each value of K from 1 to 11. The most likely number of clusters (K) was estimated with STRUCTURE HARVESTER^28^, concatenated over replicates with CLUMPP^29^, and graphically displayed with Distruct^30^. Second, we performed a Principal Component Analysis (PCA) of individual genotypes with the R packages *ade4* and *adegenet*^31^. Third, we computed pairwise genetic distance (F_st_) for populations with *n* ≥ 5 using the R package *hierfstat*^32^.

Mitochondrial DNA from 230 hybrid zone samples was profiled by enzyme restriction with *MseI*, which differentially cuts the three mitotypes (methods in^33^). In addition, we included the published mtDNA barcoding data from two additional populations located close to our loc. 15–16 (*n* = 9 *H. arborea*/*molleri* and 3 *H. meridionalis*,;^22^ see File S1).

Finally, we fitted sigmoid clines to mitochondrial frequency and nuclear admixture coefficients (STRUCTURE’s Q) along a north-south transect running along the Atlantic coast (loc. 4–12, 15–16, 21–24, 26–29), using the R package *hzar*^34^. We performed model selection between two- (width and center) to eight-parameter clines (width, center, parental frequencies, position and steepness of exponential tails), and kept the model with the best AIC score.

### *Phylogeography of W-Palearctic* Hyla

In order to provide an up-to-date and comprehensive overview of species distributions, range limits, and the extent of the ten contacts documented so far in W-Palearctic *Hyla*, we combined our new *H. arborea/molleri* population genetic data with those from 24 previously-published studies on the phylogeography and hybrid zones of this group, totaling records from 857 different localities. These included general phylogenetic/phylogeographic accounts^21,23,35^, intra-specific genetic work on *H. meridionalis* and *H. carthaginiensis*^36,37^, *H. savignyi* and *H. felixarabica*^38,39^, *H. sarda*^40,41^, *H. intermedia* and *H. perrini*^42,43^, *H. arborea*^26,44^, *H. molleri*^45,46^, and *H. orientalis*^38,39,47^, as well as targeted surveys of the transitions for *H. meridionalis*/*arborea-molleri*^22^, *H. savignyi*/*felixarabica*^48^, *H. arborea*/*perrini*^49,50^, *H. intermedia*/*perrini*^20^ and *H. arborea*/*orientalis*^33,51^. Based on the distribution of genetic diversity and ecological niche modelling, these studies also inferred the location of glacial refugia and routes of post-glacial recolonization for each species, providing insights on the relative age of contact zones that we summarized in File S2.

For divergence times, we relied on the fully-resolved phylogeny of^20^, built from genome-wide nuclear sequences (~42 kb), and a molecular clock calibrated with the tree root at ~20 Mya^52,53^, and the Messinian divergence of the Tyrrhenian *H. sarda*^21,35,54^.

*Hyla* species transitions received heterogeneous assessments in terms of methodology (numbers and nature of loci, type of analyses) and sampling schemes. Therefore, rather than a quantitative measure of hybrid zone width (which was available for only three out of the ten contacts), here we distinguished three discrete categories reflecting the type of transition. These categories were: (1) sympatry without gene flow; (2) narrow transitions (<50 km) with admixture (if detected) restricted to few localities at the contact; (3) wide transitions (>50 km) involving widespread admixture far away from the contact.

### Species distribution modelling

To complement previous knowledge on the putative age of contact zones, we built species distribution models (SDM) to predict the ecological niches and the LGM ranges for all ten *Hyla* taxa. Such analyses have been independently conducted in previous work on some taxa^20,46–48^, but here we aimed to provide comparable outcomes across all of them, as obtained from a single methodology. To this end, analyses were performed with MaxEnt 3.4.1^55^, implementing occurrence filtering, tests for distinct candidate sets of environmental variables, and multiple combinations of model parameters (feature classes, regularization multipliers, and sets of variables), using accessible areas for model calibration and multiple statistical criteria (partial ROC, omission rates, and AICc) for model selection.

A total of 6,363 localities were used, comprising our own records and previously published data (File S3), filtered to avoid spatial autocorrelation and duplication by NicheToolBox^56^. We selected localities of each species to obtain the maximum number of localities that were at least 10 km (0.093°) apart, which resulted in 3,685 presence-only locations for analyses. Altitude and 19 bioclimatic layers (30 arc seconds and 2.5 arc minutes spatial resolutions) representative of the climatic data over ~1950–2000 were extracted from the WorldClim 1.4 database (http://www.worldclim.org). An additional three layers were considered: the aridity index (Global Aridity and Potential Evapo-Transpiration; http://www.cgiar-csi.org/data/global-aridity-and-pet-database), spatial homogeneity of global habitat (EarthEnv; http://www.earthenv.org/texture.html), and the global percent of tree coverage (https://github.com/globalmaps/gm_ve_v1). To consider topography in the model, four landscape layers were calculated with QGIS: aspect, exposition, slope, and terrain roughness index. For the LGM predictions, we used the same 19 bioclimatic layers (2.5 arc minutes spatial resolution) and applied two general atmospheric circulation models: the Community Climate System Model (CCSM; http://www2.cesm.ucar.edu/) and the Model for Interdisciplinary Research on Climate (MIROC^57^). Analyses were conducted with species-specific masks covering to the area of occurrence and adjacent regions.

To eliminate predictor collinearity before generating the model, we calculated Pearsons’s correlation coefficients for all pairs of bioclimatic variables using ENMTools^58^. For correlated pairs (│r│> 0.75), we excluded the variable that appeared less biologically important for *Hyla* tree frogs. Analyses were performed separately for each species, and we ran MaxEnt models with 10 replicate bootstrap resamplings. Model calibration consisted in evaluating models created with distinct regularization multipliers (0.5 to 6 at intervals of 0.5), feature classes (resulted from all combinations of linear, quadratic, product, threshold, and hinge response types), and from two different sets of layers. The first set included altitude, the aridity index, spatial homogeneity, the percent of tree coverage, the four landscape variables, as well as the uncorrelated bioclimatic layers; the second was restricted to the most important layers, i.e. those with a relative contribution above 10% in preliminary analyses. A total of 696 candidate models were thus built for each species. The best parameter settings were selected considering statistical significance (partial ROC), predictive power (omission rates E = 5%), and complexity level (AICc) obtained using the R package *kuenm*^59^. Finally, territories covered by glaciers and seas at LGM times were cut out from the corresponding distributions. These are illustrated in File S4, based on available data^60,61^.

## Results

### Hyla arborea/molleri *hybrid zone*

**Figure 1:**
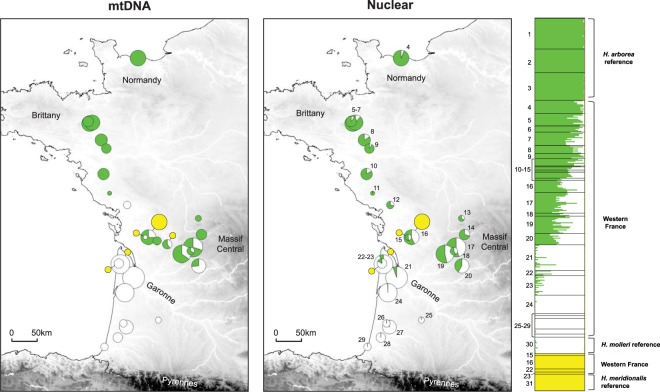
Population genetics of the *H. arborea*/*molleri*/*meridionalis* contact zone in Western France. Proportion of each mtDNA haplotype (left) and the average admixture coefficient of each population based on 26 microsatellites (right); barplots show individual admixture coefficient (STRUCTURE) for the study area and reference samples; pie charts are proportional to sample sizes. The maps were created in QGIS 3.4 (https://qgis.org).

**Figure 2:**
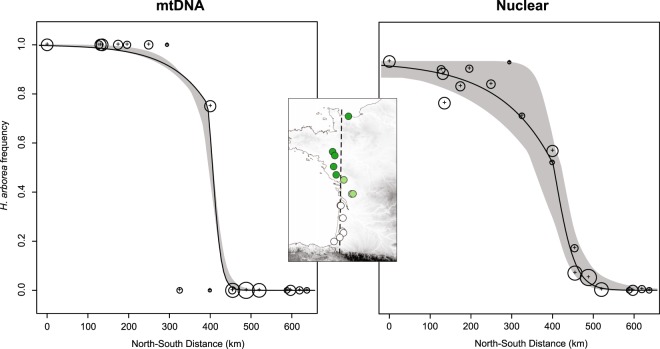
Cline analysis of mitochondrial frequency (left) and nuclear genome average (right) across the *H. arborea*/*molleri* hybrid zone along a north-south geographic transect (shown on the inset map); for both datasets, the selected cline model involved asymmetric introgression tails, which were larger on the *H. arborea* side; encircled crosses illustrate sample size for each population; shaded areas represent the 95% confidence interval of fitted clines.

Our data supported widespread nuclear admixture between *H. arborea* and *H. molleri*, spanning from the Garonne basin (loc. 21) to the Massif Central (east of loc. 14) and Normandy (loc. 4) (Fig. 1). The mitochondrial picture was broadly concordant, with mitotypes shared in several localities north of the Garonne (loc. 15–20). Clines were wide (width *w* = 98 km for nuclear loci; *w* = 43 km for mtDNA), remarkably coincident (center *c* = 407 km for nuclear loci; *c* = 406 km for mtDNA), and both bear a long introgression tail on the *H. arborea* side (the selected cline model under the AIC criterion, Fig. 2). There was no trace of mitochondrial or nuclear introgression with the sympatric and syntopic *H. meridionalis* (Fig. 1). The PCA (File S5) and pairwise F_st_ (File S6) disentangled the three gene pools and confirmed that individuals collected in Western France represent the entire *H. arborea*–*molleri* spectrum.

### Species transitions

**Figure 3:**
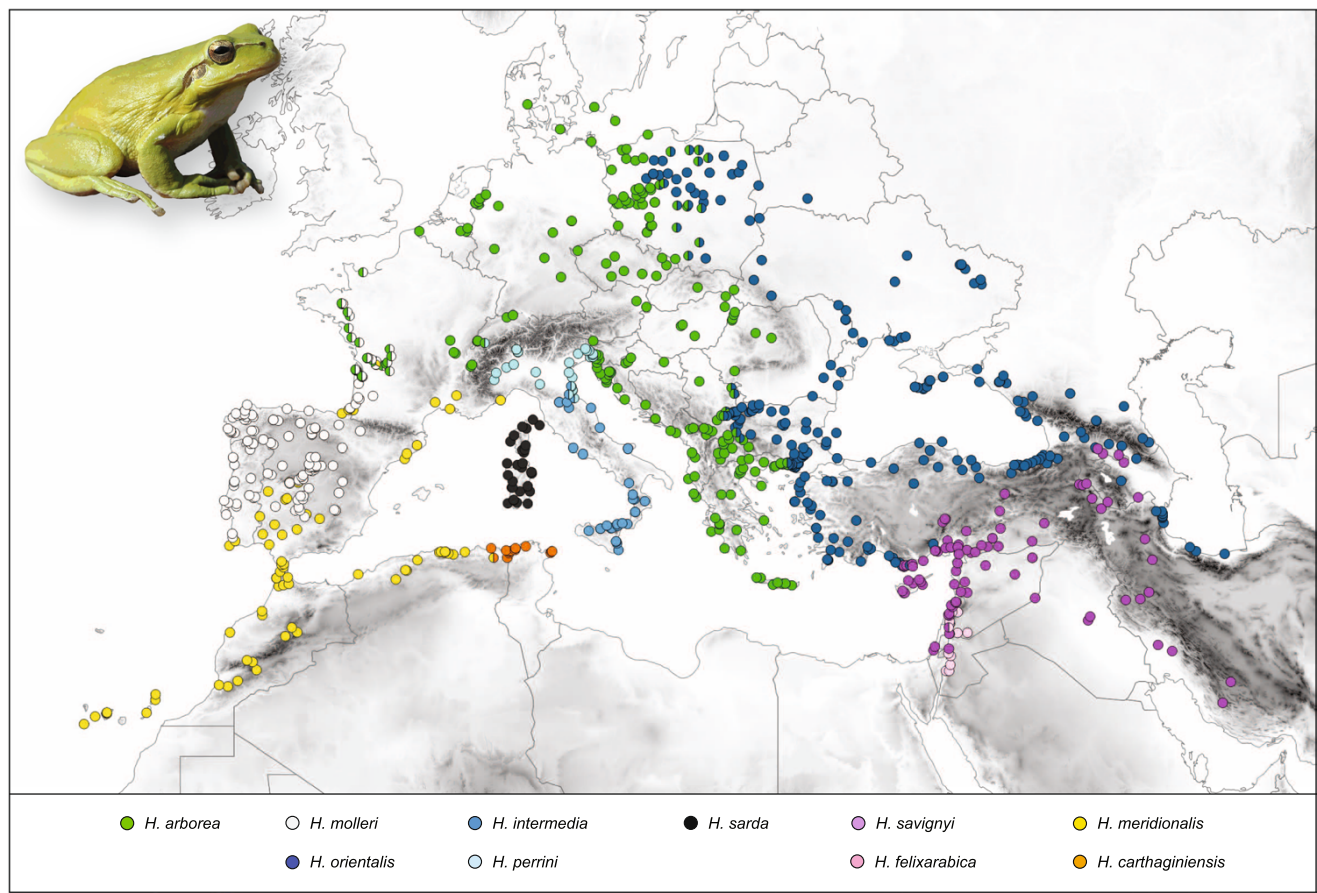
Distribution of W-Palearctic tree frogs based on genetically barcoded populations, combining 25 studies. At transitions, populations were considered as a mix (represented in half circles) if they featured both mtDNA haplotypes and/or nuclear genotypes consistent with admixture (STRUCTURE’s Q < 0.9 in multilocus analyses; co-segregation of species-diagnostic alleles). The map was created in QGIS 3.4 (https://qgis.org). Photo: *Hyla meridionalis* from southeastern France, by CD.

The distribution of W-Palearctic *Hyla* taxa is shown in Fig. 3, based on genetic barcoding from 25 studies (including the present paper). A summary of species interactions and the corresponding literature is provided in Table 1 and File S2, from which we categorized the following. (1) One case of sympatry without gene flow despite occasional hybridization: *H. meridionalis/arborea-molleri*; (2) Five steep parapatric transitions, with introgression (if any) restricted at range margins, and narrow cline width (<50 km): *H. savignyi/felixarabica* in N-Israel; *H. arborea*/*perrini* in NE-Italy; *H. meridionalis*/*carthaginiensis* in NE-Algeria; *H. arborea*/*orientalis* in the Balkans; *H. savignyi*/*orientalis* in S-Turkey, where evidence for hybridization/admixture is presently lacking. (3) Three wide continuous transitions with large nuclear clines (>50 km) and admixture over more than a hundred kilometers: *H. arborea*/*orientalis* in Poland; *H. arborea*/*molleri* in SW-France; *H. intermedia/perrini* in Central Italy; the *H. arborea/perrini* hybrid swarm from W-Switzerland was also assigned to this category. Considering the ten contacts altogether, the relationship between the amount of admixture and the divergence time was significant (ordinal logistic regression: LR χ^2^ = 7.72, *n* = 10, df = 1, *P* = 0.005).

**Table 1:** Summary of ten secondary contact zones among W-Palearctic tree frogs, in respect to the divergence time between species pairs (Div. in Ma; taken from^20^; nodes are labelled in Fig. 6), the putative epoch of first contact based on previous studies and past species distribution modelling (see File S2), and the type of transition (see File S2, Fig. 3 and the listed references), with the corresponding category in our meta-analysis (^a^sympatry without gene flow, ^b^steep transition, ^c^wide transition/hybrid swarm); *w*: hybrid zone width, as measured from cline analyses of transects.

Node	Region	Species pair	Div.	Contact	Transition	Reference
A	W-France	*H. meridionalis/arborea- molleri*	20	Antiquity	sympatry without gene flow^a^	^22^
B	S-Anatolia	*H. orientalis*/*savignyi*	8.8	≥LGM	steep, hybridization/admixture not detected^b^	^38,39^
C	NE-Italy/Slovenia	*H. arborea*/*perrini*	6.6	≥LGM	steep, faint traces of admixture^b^	^49^
C	W-Switzerland	*H. arborea*/*perrini*	6.6	1950s	hybrid swarm^c^	^50^
D	Balkans	*H. arborea/orientalis*	5.1	≥LGM	steep (*w* = 30–32 km)^b^	^33^
D	Poland	*H. arborea/orientalis*	5.1	post-glacial	wide, admixture across a largearea (>200 km)^c^	^51^
D	W-France	*H. arborea/molleri*	5.1	post-glacial	wide (*w* = 98 km)^c^	this study
E	Central Italy	*H. perrini*/*intermedia*	3.5	post-glacial	wide (*w* = 96 km)^c^	^20^
F	Levant	*H. savignyi*/*felixarabica*	6.4	≥LGM	steep, geographically restricted admixture^b^	^38,48^
G	E-Algeria	*H. meridionalis/carthaginiensis*	4.1	≥LGM	steep, admixture detected at a single locality^b^	^37^

### LGM distributions and relative ages of the species transitions

**Figure 4:**
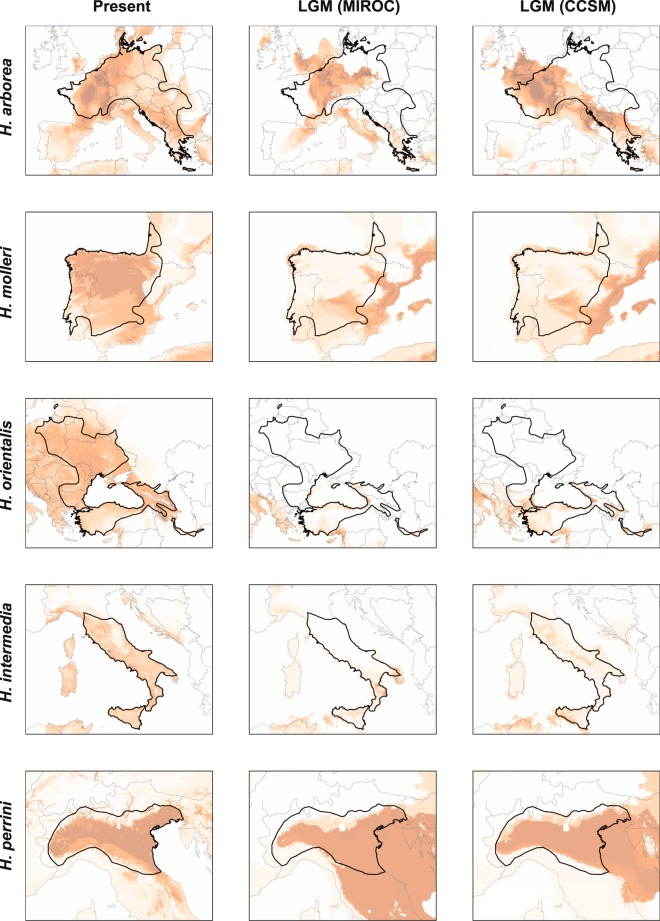
Projected distributions under present and LGM conditions (MIROC and CCSM scenarios) for European *Hyla* species. Color warmth (from white to brown) reflects the probability of occurrence. Dark lines show current species ranges. The maps were created in QGIS 3.4 (https://qgis.org).

**Figure 5:**
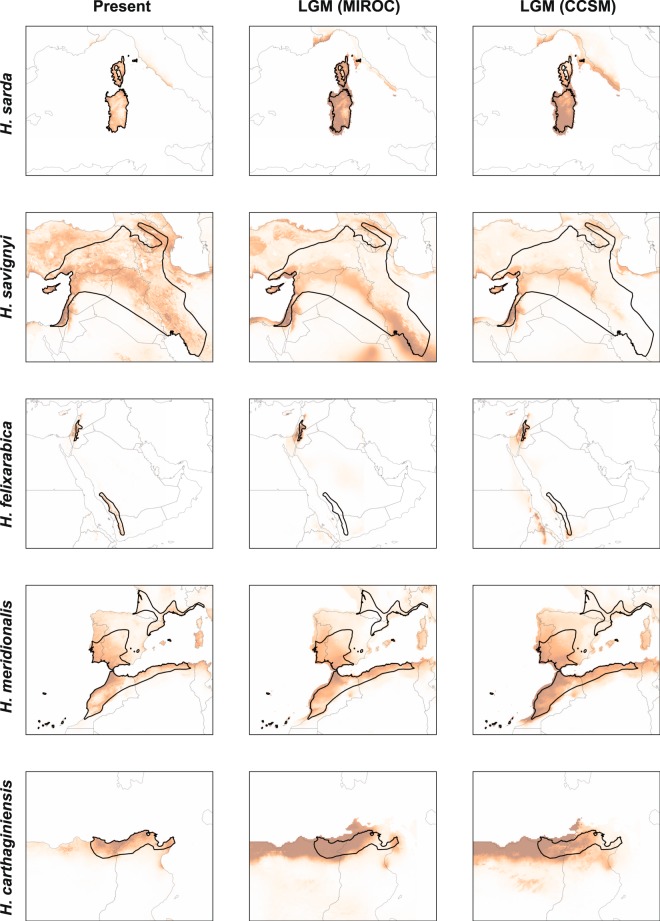
Projected distributions under present and LGM conditions (MIROC and CCSM scenarios) for Tyrrhenian, Middle-Eastern and N-African *Hyla* species. Color warmth (from white to brown) reflects the probability of occurrence. Dark lines show current species ranges. The maps were created in QGIS 3.4 (https://qgis.org).

All species distribution models (SDM) received very good fits, indicative of robust predictions (File S7). Projected distributions for each species are shown in Figs. 4 and 5. In Europe, LGM conditions appeared unsuitable across the northern parapatric ranges of *H. arborea* and *H. orientalis*, but were more favorable in the Balkans (Fig. 4). For *H. molleri*, the models identified hospitable glacial ranges along the coasts (Fig. 4). In Italy, the probabilities of occurrence remained high for *H. perrini*, but not for *H. intermedia*, which appeared scattered throughout the Peninsula (Fig. 4). In N-Africa, the Maghreb coast offered favorable conditions for both *H. meridionalis* and *H. carthaginiensis* (Fig. 5). Finally, the present and LGM predictions were globally similar for the insular *H. sarda*, and the Middle-Eastern *H. savignyi* and *H. felixarabica* (Fig. 5).

**Figure 6:**
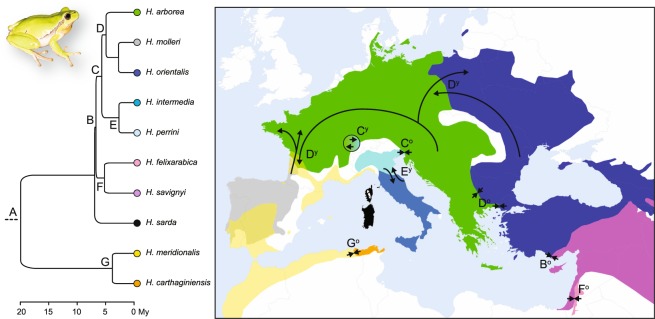
Phylogeny and distribution of Western-Palearctic tree frogs, illustrating post-glacial expansions and the extent of introgression in secondary contact zones in respect to their amount of divergence (capital letters: phylogenetic nodes) and age since contact (subscripts: “young” as post-LGM (y) or “old” as pre-LGM (o)). Species distributions are adapted from^77^. See Table 1 for details on the patterns of admixture and relevant references. The map was created in QGIS 3.4 (https://qgis.org). Photo: *H. arborea* from southeastern France, by CD.

**Figure 7:**
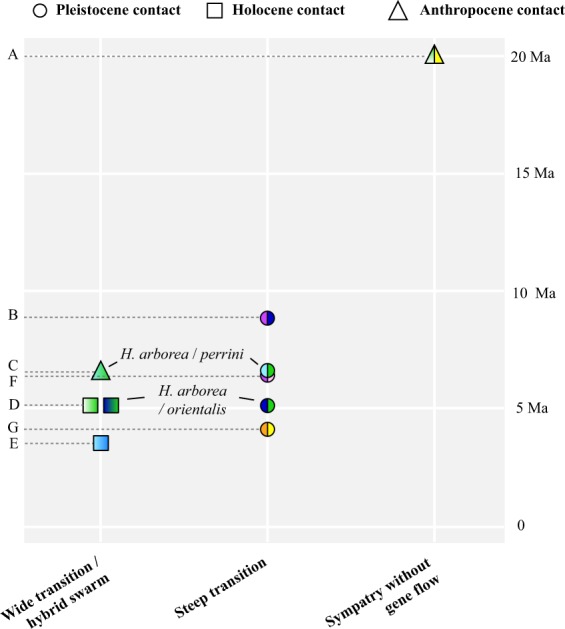
Species transitions in respect to divergence time in W-Palearctic tree frogs. Color codes refer to Figs. 3 and 6, and replicate contacts between the same species are specifically labelled. Letters indicate the phylogenetic nodes shown in Fig. 6. Symbols differentiate the contacts according to when they were putatively formed.

Integrating the projected species distributions with the well-documented phylogeography of W-Palearctic *Hyla* (File S2), the wide transitions/hybrid swarms were all presumably formed after the LGM (Holocene or Anthropocene contact): *H. arborea*/*molleri*, *H. arborea*/*orientalis* in Poland, *H. intermedia*/*perrini*, *H. perrini*/*arborea* in W-Switzerland (Fig. 6). In contrast, the steep transitions all encompass putative glacial refugia (Pleistocene contact): *H. savignyi/orientalis*: *H. arborea/orientalis* in the Balkans; *H. arborea*/*perrini*; *H. savignyi/felixarabica*; *H. meridionalis*/*carthaginiensis* (Fig. 6). Finally, the sympatric (but not admixing), early-diverged pair *H. meridionalis*/*arborea*–*molleri* result from a human introduction (File S2). Accordingly, the relative age of contact (Holocene/Anthropocene *vs* Pleistocene) was a significant predictor of these three categories of transition (wide, steep, or sympatry without gene flow), based on an ordinal logistic regression (LR χ^2^ = 3.85, *n* = 10, df = 1, *P* = 0.049), and as illustrated in Fig. 7. The effect was also significant when comparing only wide *vs* steep hybrid zones with a χ^2^ test of independence (χ^2^ = 5.41, *n* = 9, df = 1, *P* = 0.020) – two categories where species pairs do not significantly differ in divergence times (Mann-Whitney-Wilcoxon test, *n* = 9, W = 13.5, *P* = 0.45).

## Discussion

### A wide tree frog transition across W-France

The widespread introgression between *H. arborea* and *H. molleri* documented across W-France confirms previous studies that reported hybrid specimens in several scattered localities^21–23^. Their genomes are thus mostly compatible, so selection against hybrids is probably weak (see below). The phylogeographic history of these two species suggests a somewhat classic scenario of post-glacial contact. According to our predicted distributions (Fig. 4, see also^46^), the Iberian *H. molleri* could have expanded from some Atlantic refugia into western France (where private mtDNA haplotypes are found^21,46^), while *H. arborea* arrived from the more distant Balkan refugium^26^. Subsequently, these two species introgressed massively along the Atlantic coast, but the dry Garonne valley may nowadays act as an effective bioclimatic barrier preventing northwards dispersal of *H. molleri* (as in other biota^62^). This hypothesis is supported by the asymmetric transition (Fig. 2): *H. arborea* allele frequencies decrease continuously with latitude, up to a sharp drop between the northern (loc. 4–20) and southern sides of the valley (loc. 21–29). Hence, the hybrid zone is probably not stable, and *H. arborea* might eventually fully backcross the hybrid populations over time. This particular scheme of hybrid zone movement is somewhat reminiscent of the *Bufotes viridis*/*balearicus* green toad transition in Italy, where hybrids became isolated north of the Po River, and cannot escape inflocks of *B. viridis* expanding southward^63^. Here, the *H. arborea*/*molleri* species pair adds a valuable point to our comparative framework of admixture *vs* history of secondary contacts.

### Tree frogs admix freely in young hybrid zones, but not in old ones

The dense phylogeographic framework, including ten contacts from the fully-resolved radiation of W-Palearctic tree frogs, supports that the width of species transitions drastically differs depending on when these were first established (Figs. 6 and 7, Table 1). Lower-Miocene lineages do not introgress (node A), as expected given the deep genetic (and phenotypic) divergence. However, Upper Miocene (nodes B–D and F) and Pliocene (nodes E–G) taxa admix significantly more across post-glacial and introduced ranges than in refugial areas (Figs. 6 and 7). Note that the divergence times reported here (from^20^, see Methods) should be taken with caution, since the genus *Hyla* might be younger than previously assumed^64^.

Because they feature hybrid zones involving the same pair, or between species that coalesced at the same time, two nodes are of particular interest for direct comparisons. First, we highlighted striking differences among the *H. arborea*/*orientalis* contacts (node D). In their Balkan refugium, mtDNA and nuclear transitions do not exceed 30 km^33^, while they extend over hundreds of kilometers in the Polish post-glacial ranges^51^. The latter mirrors the wide post-glacial hybrid zone documented herein for *H. arborea*/*molleri*, which share the same amount of divergence (node D). Second, the slightly older *H. arborea* and *H. perrini* (node C) do not admix along their natural margins in NE-Italy/Slovenia^49^, where subrefugia of both species are putatively located^20,26^ (see File S2). Nevertheless, this pair widely hybridized in W-Switzerland^50^ following introductions of *H. perrini* in the 1950s^65^. All other tree frogs meeting in ecologically stable regions featured steep mitochondrial and nuclear transitions (File S2), i.e. *H. savigny*/*orientalis* in southern Anatolia (note B^38,39^), *H. savignyi*/*felixarabica* in the Levant (node F^38,48^, and *H. meridionalis*/*carthaginiensis* in the E-Maghreb (node G^37^). In Italy, the *H. intermedia*/*perrini* contact is presumably post-glacial (see details in File S2), but there the wide transition may also result from the weak reproductive barriers between these recently-diverged taxa (~3.5 Ma^20^).

Different opportunities for dispersal would hardly explain these contrasting species transitions. European tree frogs are cosmopolitan and some of the most mobile amphibians (up to 12 km movements^66,67^). While large rivers and their valleys were identified as contemporary geographic barriers, this involves both refugial and post-glacial transitions, e.g. the Vistula between *H. arborea* and *H. orientalis* in Poland^51^; the Garonne between *H. arborea* and *H. molleri* (this study); the Isonzo between *H. arborea* and *H. perrini*^49^; the Jordan Valley between *H. savignyi* and *H. felixarabica*^38,48^. Reciprocally, no geographic barriers were identified for some narrow transitions, e.g. *H. meridionalis*/*carthaginiensis*^37^, *H. arborea*/*orientalis* in the Balkans^33^. Ecological barriers also appear unlikely, given that the projected distributions under current conditions remarkably overlapped between parapatric species, notably at their transitions (Figs. 4 and 5).

Several limitations prevent a more quantitative assessment of the relationship between the time since contact and the patterns of introgression across the radiation, which would strengthen our results. First, the heterogeneity of the hybrid zone datasets published on *Hyla* (uneven sampling, diverse types of molecular markers) precludes comparable and spatially-explicit genetic reconstructions of the demographic history of hybridizing populations (e.g.^68^), which could have provided estimates of the ages since first contact. Genomic phylogeographic data (e.g. RAD-seq) and transect sampling for all hybrid systems would have also allowed to compare admixture patterns with more accuracy. Second, our categorization of the relative ages of contact, taken from phylogeographic data and SDM predictions, assumes that species niches did not change since the LGM, and that old hybrid zones remained static through time. Accordingly, it is possible that some contact zones located within southern glacial refugia were established post-LGM following regional expansions from separate micro-refugia, even if they seem connected by suitable LGM conditions in our analyses. Yet, these southern contacts should still be older than the Holocene northern contacts located thousands of kilometers away from their source populations. Third, and while we assume that local geographic barriers played a minor effect on the patterns of introgression (see above), this remains to be thoroughly addressed by habitat analyses, notably in the context of landscape changes during the Anthropocene. In parallel, the relative contribution of pre- *vs* post-zygotic isolation in limiting admixture across the narrow hybrid zones is unclear; characterizing pre-mating barriers (breeding calls, pheromones) between supposedly cryptic incipient species remains a major gap in the amphibian literature. Accounting for all the factors potentially affecting the age and admixture patterns at secondary contact zones is already colossal for a single transition, and it becomes logistically impossible in a comparative framework involving multiple contacts.

While acknowledging these limitations, the qualitative data in hand thus tend to support our working hypothesis that the contrasted hybrid zones in *Hyla* partly result from the phylogeographic dynamics that led to their establishment. Compared to refugial areas, colonizers at the front of post-glacial expansions should hybridize more with related species^69^, due to limited mate choice, multiple contacts across unsettled parapatric ranges, and few opportunities to evolve reinforcement given the much shorter time since first contact. While the prevalence of reinforcement in the wild is debated^15^, this mechanism is known to contribute to speciation in hylid frogs^70^. For the same species pair, different genomic backgrounds (i.e. intraspecific lineages) may also affect patterns of incompatibilities and admixture; here this could apply to the two *H. arborea*/*orientalis* contacts, which involve independent refugial lineages^26,51^. Therefore, replicate hybrid zone analyses appear necessary to fully appreciate where two taxa lay on the speciation continuum.

In this context, our findings have profound consequences for species delimitation. Phylogeographic lineages are increasingly considered in taxonomy^8^, and when possible, their specific or subspecific rank may be decided based on whether they are (partially) reproductively isolated, following the universal biological species concept^3^. Accordingly, wide transitions are interpreted as a lack of reproductive barriers, as opposed to narrow transitions^5,71^. An ad hoc way to quantify reproductive isolation from hybrid zones is to calculate selection against hybrids from cline width *w* and dispersal *σ*, with the equations of^72^. Without extrinsic selection (as expected for ecologically-similar cryptic species), the coefficient s* corresponds to the fitness difference between populations from the center and the edge of the hybrid zone (under heterozygote disadvantage), given *w* ≈ *2σ*/√s*^72^. In *Hyla*, taking a dispersal rate σ ~ 0.5 km/year (adapted from^66^, who found average movements of 1.5 km over a three year capture-mark-recapture survey), and average ages at maturity and longevity of 2 and 6 years, respectively (reviewed by^73^), the refugial contact between *H. arborea* and *H. orientalis* (*w* = 30 km^33^,) reflect ten-fold higher estimates (s* = 0.05) than the post-glacial contact between *H. arborea* and *H. molleri* (s* = 0.005, from *w* = 98 km), despite similar genetic divergence. The extent of admixture between incipient species might thus misguide taxonomic conclusions if the history of contacts is not considered. When possible, the grey zone of speciation would be best inferred from refugial areas, where selection had time to operate and species transitions putatively reached an equilibrium. Reciprocally, narrow post-glacial hybrid zones would strongly indicate incipient speciation. To conclude, we recommend that taxonomists interpret introgression patterns at range margins in the light of two key timings of speciation, i.e. the time of divergence and the time since first contact.

Finally, the present results call for caution when assessing the hybridization potential of invasive species. Genetic pollution is a major issue of bioinvasions^74,75^, and the risk should not be underestimated even if the local and invasive species rarely hybridize in natural ranges. This is well-illustrated here by the introduction of *H. perrini* north of the Alps, which extensively admixed with local *H. arborea*^50^, despite nearly absent introgression at their natural phylogeographic transition^49^. In the syntopic fishes *Alosa pseufoharengus* and *A. aestivalis*, human-driven habitat fragmentation disrupted their well-established reproductive barriers, leading to whole-hybrid populations^76^. If the diverged genomes of incipient species are partly incompatible, such “forced” hybridization can severely affect the sustainability of local taxa, and thus precipitate their extinctions.

## Conclusions

Combining new data with fifteen years of research on the W-Palearctic radiation of tree frogs (*Hyla*), we emphasized how admixture patterns at species transitions can drastically vary depending on their biogeographic history. For similar evolutionary divergence, species pairs form wider hybrid zone in post-glacial and introduced ranges, compared to regions spanning their putative glacial refugia. We thus conclude that both the divergence time and the time since contact significantly affect the extent of admixture between incipient species. Future studies should focus on identifying the proximate mechanisms, and especially test whether reinforcement evolved in long-term contacts. Informed taxonomic decisions are thus best taken from replicate hybrid zone analyses that account for the late-Quaternary dynamics of phylogeographic transitions.

## Supplementary information

Supplementary Information.

Supplementary Information2.

## Data Availability

The data of this study is available as supplementary information.
