# Supplementary material for: The effect of phylogeographic history on species boundaries: a comparative framework in Hyla tree frogs
Source: Sci Rep. 2020 Mar 26;10:5502. doi: 10.1038/s41598-020-62382-4 (PMC7099067; doi:10.1038/s41598-020-62382-4)
Supplement: Supplementary file 2 — Supplementary Information2. [file 41598_2020_62382_MOESM2_ESM.pdf]

SCIENTIFIC REPORTS

Supplementary Information for

The effect of phylogeographic history on species boundaries: a comparative framework in *Hyla* tree frogs

Christophe Dufresnes, Matthieu Berroneau, Sylvain Dubey, Spartak N. Litvinchuk, Nicolas Perrin

Content **page**

File S1: Details on the populations analyzed in W-France 2

File S2: Accounts on glacial refugia and contact zones in W-Palearctic *Hyla* 3

File S3: Occurrence records of W-Palearctic *Hyla* 9

File S4: Glacial extent and coastlines during the LGM 10

File S5: PCA of the W-France hybrid zone 11

File S6: NJ tree of pairwise genetic distance in the W-France hybrid zone 12

File S7: Performance of the SDMs and variable contributions 13

File S8: Archive of the data associated to this study -

File S1: Details on the populations analyzed to study the *H. arborea* / *molleri* hybrid zone.

ID	Species	Locality	Latitude	Longitude	*n* nuclear	*n* mtDNA
1	*H. arborea* (reference)	Switzerland, Vaud, Lavigny	46.50	6.42	30	-
2	*H. arborea* (reference)	Netherlands, Barchem	52.12	6.48	23	-
3	*H. arborea* (reference)	France, Bourgogne, Chaseux	46.52	4.72	27	-
4	*H. arborea* / *molleri*	France, Normandie, Arromanches	49.33	-0.63	13	13
5	*H. arborea* / *molleri*	France, Bretagne, Bréal-sous-Montfort	48.05	-1.87	12	12
6	*H. arborea* / *molleri*	France, Bretagne, Monterfil	48.07	-1.98	6	6
7	*H. arborea* / *molleri*	France, Bretagne, Saint-Thurial	48.02	-1.94	13	13
8	*H. arborea* / *molleri*	France, Pays-de-la-Loire, Lusanger	47.70	-1.62	8	8
9	*H. arborea* / *molleri*	France, Pays-de-la-Loire, Notre-Dame-des-Langueurs	47.52	-1.48	5	5
10	*H. arborea* / *molleri*	France, Pays-de-la-Loire, Les Grimaudières	47.01	-1.55	7	7
11	*H. arborea* / *molleri*	France, Pays-de-la-Loire, Le Chaigneau	46.63	-1.38	1	1
12	*H. arborea* / *molleri*	France, Pays-de-la-Loire, Velluire	46.40	-0.90	3	3
13	*H. arborea* / *molleri*	France, Nouvelle-Aquitaine, Bellac	46.13	1.01	2	2
14	*H. arborea* / *molleri*	France, Nouvelle-Aquitaine, Bournazaud	45.81	1.09	6	6
15	*H. arborea* / *molleri* - *H. meridionalis*	France, Nouvelle-Aquitaine, Les Chaudroles	45.75	-0.42	2 (2)	1 (2)
16	*H. arborea* / *molleri* - *H. meridionalis*	France, Nouvelle-Aquitaine, Cherves-Richemont	45.75	-0.33	12 (13)	12 (13)
17	*H. arborea* / *molleri*	France, Nouvelle-Aquitaine, Mazaubrun	45.55	0.87	20	20
18	*H. arborea* / *molleri*	France, Nouvelle-Aquitaine, Maumont	45.47	0.80	3	3
19	*H. arborea* / *molleri*	France, Nouvelle-Aquitaine, Saint-Félix-de-Bourdeilles	45.41	0.57	17	17
20	*H. arborea* / *molleri*	France, Nouvelle-Aquitaine, Ajat	45.18	1.01	11	11
21	*H. arborea* / *molleri*	France, Nouvelle-Aquitaine, St-Aubin-de-Médoc	44.94	-0.73	25	25
22	*H. arborea* / *molleri* - *H. meridionalis*	France, Nouvelle-Aquitaine, Hourtin	45.23	-1.11	5 (3)	5 (3)
23	*H. arborea* / *molleri* - *H. meridionalis*	France, Nouvelle-Aquitaine, Cartignac	45.21	-1.08	19 (2)	19 (2)
24	*H. molleri*	France, Nouvelle-Aquitaine, Facture	44.64	-0.95	18	18
25	*H. molleri*	France, Nouvelle-Aquitaine, Losse	44.10	-0.06	2	2
26	*H. molleri*	France, Nouvelle-Aquitaine, Sindères	44.03	-0.99	3	3
27	*H. molleri*	France, Nouvelle-Aquitaine, Rion	43.96	-0.91	10	10
28	*H. molleri*	France, Nouvelle-Aquitaine, St-Paul-lès-Dax	43.74	-1.13	5	5
29	*H. molleri*	France, Nouvelle-Aquitaine, Ondres	43.56	-1.48	3	3
30	*H. molleri* (reference)	Spain, Castilla, Fresnedilla	40.21	-4.65	16	-
31	*H. meridionalis* (reference)	France, Provence-Alpes-Côtes-d'Azur, La Tour du Valat	43.52	4.70	16	-
BAS	*H. arborea* / *molleri*	France, Nouvelle-Aquitaine, Bassac	45.68	-0.09	-	4
PUY	*H. arborea* / *molleri*	France, Nouvelle-Aquitaine, Puymoyen	45.61	0.18	-	5

File S2

Phylogeography and contact zones in W-Palearctic *Hyla* – a summary

The following briefly reports on patterns of diversity and distribution in each W-Palearctic tree frog, based on available phylogeographic evidence, with a particular focus on the extent of admixture at species transitions and the relative age of contacts.

Hyla arborea

PHYLOGEOGRAPHY IN BRIEF – Detailed multilocus accounts evidenced three Pleistocene refugia throughout the Balkan Peninsula, namely in southern Greece, the Pannonian Basin and the Adriatic coast [1-2]. Northern and Western European populations belong to the Pannonian clade, which exhibit signs of recent demographic and spatial expansions [1, 3]. Mitochondrial and nuclear diversity significantly decreased with distance from the Balkans, as expected with founder effects during post-glacial recolonization [1-2]. LGM conditions were accordingly suitable in the Balkans, especially along the Adriatic and Greek coastlines (Fig. 4). Northeastern Europe was accordingly unhospitable, but the climatic models did feature high probabilities of occurrence in northwestern Europe. Yet, and while private mtDNA haplotypes were found in the latter [1], these are weakly differentiated from the Balkan populations, and thus do not support an additional northern refugium. To conclude, it is very likely that *H. arborea* survived the Quaternary glaciations in the Balkan Peninsula and colonized northern and western Europe after the LGM.

CONTACT ZONES – **(1) *H. arborea/perrini* in N-Italy / Slovenia.** Narrow transition partially mediated by the Izonso River at the border between Slovenia and Italy [4]. Considered as a Pleistocene contact given that the area overlaps putative refugial ranges of both species, according to SDM analyses (Fig. 4, see [5] for *H. perrini*, and below) and high levels of genetic diversity (see [1] for *H. arborea*). Verardi and colleagues [4] analyzed this contact using diagnostic mtDNA and allozyme markers and found mostly pure individuals, expect for a few that exhibited very limited admixture (<5%). **(2) *H. arborea/perrini* in W-Switzerland.** Anthropocene contact resulting from the introduction of *H. perrini* in the 1950s by wildlife enthusiasts, within the natural range of *H. arborea* [6]. Multilocus analyses revealed that both species massively admixed and formed a hybrid swarm, where no pure individuals remain [7]. **(3) *H. arborea/orientalis* in the Balkans.** Narrow transitions with geographically restricted admixture along the Aegean coast (northern Greece) as well as the Balkan and Rhodope slopes, despite no dispersal barriers (CD and A. Brelsford pers. obs.). Considered as Pleistocene contacts since they encompassed putative refugia of both species, according to genetic [1, 8] and SDM evidence (Fig. 4, see also [8] for *H. orientalis*). Cline analyses along two transects inferred hybrid zone widths of 30km in southeastern Serbia (nearby Niš) and 32km in northeastern Greece (Thrace) for microsatellite loci [9]. Mitochondrial DNA had more variable widths (39km and 6km, respectively; [9]). **(4) *H. arborea/orientalis* in N-Europe**. In Poland, *H. orientalis* reaches the confluence of the Vistula and Notec rivers in the west, and nuclear and mitochondrial introgression was documented over about 200km along the Baltic coastline (Gdansk area, [10]). Several hybrid populations were also found in central (Lodz area) and southern Poland (Krakow area; see also [11]). Considered as an Holocene contact as northern Europe was recolonized by both species after the LGM, as supported by inferences of population expansions [1, 3, 8], low genetic diversity, and unsuitable conditions predicted by SDM analyses (Fig. 4, see also [8] for *H. orientalis*). **(5) *H. arborea/molleri*.** Wide hybrid zone

spanning across W-France with admixture detected over 400km from the Garonne River to Normandy (Fig. 1). Considered as a Holocene contact given that *H. arborea* colonized the region from the Balkans after the LGM, which is supported by signature of demographic expansions and patterns of genetic diversity [1-3]. Cline analyses along the Atlantic coastline suggested widths of 98km for nuclear loci (microsatellites) and 42km for mitochondrial DNA (Fig. 2), centered between Bordeaux and Nantes, but with extensive admixture on the *H. arborea* side.

Hyla orientalis

PHYLOGEOGRAPHY IN BRIEF – The phylogeography of *H. orientalis* is detailed in [8], who identified multiple Pleistocene lineages distributed around the Black Sea, southwestern Anatolia, the Caucasus and Hyrcania (southern Caspian region). According to lineage distribution, diversity and SDM analyses, the Black Sea and Caucasian / Hyrcanian lineages probably persisted in small scattered refugia [8]. Northeastern populations (Russia, Ukraine, Belarus and Poland) result from post-glacial expansions of the western Black Sea clade [3, 8]. In contrast, LGM conditions were milder along the Mediterranean coastlines in western and southern Anatolia (as well as nearby Greece), suggesting that the distribution of *H. orientalis* was probably continuous throughout the late-Quaternary (Fig. 4, [8]).

CONTACT ZONES – **(1) *H. orientalis/savignyi*.** Parapatric ranges extending from southern Anatolia to the Caucasus and the Caspian Sea. Considered as Pleistocene contacts given the suitable conditions in the regions throughout the late-Quaternary, according to SDM analyses (Fig. 4–5, see also [8] and [12] for *H. orientalis* and *H. savignyi*, respectively). Mitochondrial surveys showed a sharp geographic transition but did not find the species' mitotypes in syntopy, despite proximity along the Anatolian diagonal and the lesser Caucasus [13-14]. The best documented contact is located along the S-Anatolian coast between Alanya and Anamur. No hybrids have been found so far – the claim by Stöck and colleagues [3] for an hybrid at Gazipasa, Turkey, is erroneous, as this individual only bears *H. orientalis* nuclear and mitochondrial alleles (see their Fig 3 and sequences deposited on GenBank). A comprehensive analysis of this transition based on multilocus nuclear data is presently lacking, so hybridization/admixture cannot be excluded. **(2) *H. orientalis/arborea.*** See the accounts for *H. arborea*.

Hyla molleri

PHYLOGEOGRAPHY IN BRIEF – Sánchez-Montes and colleagues [15] identified separate genetic groups throughout Iberia that likely represent independent Pleistocene shelters. Regional admixture among these groups led to heterogeneous levels of genetic diversity between regions (see also a previous mitochondrial study: [16]). French populations are closely related to Galician ones (NW-Spain), but bear private alleles [3, 15]. LGM conditions were suitable across most Iberia [15], especially along the Atlantic and Mediterranean coasts, and possibly near the corridor that connects the Spanish and French populations (Fig. 4).

CONTACT ZONES – **(1) *H. molleri/arborea*.** See the account of *H. arborea*. **(2) *H. molleri/meridionalis.*** In parts of its range (including the *H. arborea*/*molleri* transition), *H. molleri* shares habitats with the north-African *H. meridionalis*, which is presumed to have been introduced during the Antiquity (Anthropocene contact, [17]). The two species, which feature different color patterns and breeding calls, may exceptionally hybridize, but the resulting progeny is sterile [18], and the sympatric populations accordingly lack any sign

of introgression (Fig. 1; [19]). Only male hybrids were documented, with intermediate breeding calls that may be avoided by females of either parental species [18-19].

Hyla intermedia

PHYLOGEOGRAPHY IN BRIEF – This species forms genetically differentiated mitochondrial and nuclear clades between southern (Calabria, Sicily) and central Italy [5, 20]. For the latter, suitable LGM conditions are scattered across the Peninsula (Fig. 4, [5]), and strong genetic signatures of demographic expansions were recovered [20].

CONTACT ZONE – **(1) *H. intermedia/perrini***. Large continuous transition along a lowland corridor between the Apennine and the Adriatic coast in Central Italy (Romagna–Ferrara). The genome-wide nuclear cline reached 96km of width, while the mtDNA was narrower (40km) [5]. There are no geographic barriers for tree frog dispersal in this region, but the contact might represent an ecological transition, since the predicted distributions under present conditions of these species show little overlap [5] (Fig. 4). Considered as a Holocene contact since *H. intermedia* was predicted to be absent in the area (Fig. 4, see [5]).

Hyla perrini

PHYLOGEOGRAPHY IN BRIEF – Mitochondrial and genome-wide nuclear analyses confirmed a single lineage [5, 20], showing strong signs of post-glacial expansions [20]. Here, the SDM analyses showed large suitable areas for *H. perrini* during the LGM (Fig. 4). In a previous study, LGM ranges were predicted across several pockets along the Alps and the north Adriatic coast (see Fig. 6 in [5]).

CONTACT ZONES – **(1) *H. intermedia/perrini***. See the account of *H. intermedia*. **(2) *H. arborea/perrini*.** See the account of *H. arborea*.

Hyla sarda

PHYLOGEOGRAPHY IN BRIEF – Molecular analyses reported several closely-related lineages on Sardinia, one of which colonized Corsica, Capraia and Elba, following population expansions during the last glacial stage, as sea level dropped [21]. Our species distribution modelling also confirmed suitable habitats throughout the Tyrrhenian archipelago during the LGM, as well as oversea dispersal routes of glacial colonization (Fig. 5).

CONTACT ZONE – This insular species does not naturally meet other *Hyla* taxa.

Hyla savignyi

PHYLOGEOGRAPHY IN BRIEF – A widespread species composed of two mitochondrial clades of little nuclear differentiation, according to available data [13]. One is mostly restricted to the Levant, while the other occupies Cyprus and most of Anatolian, Caucasian and Euphrates ranges [13-14]. SDM analyses identified wide areas potentially occupied by *H. savignyi* during the LGM, including the southern Anatolian coast and the Levant (Fig. 5).

CONTACT ZONES – **(1) *H. orientalis*/*savignyi*.** See the account for *H. orientalis*. **(2) *H. savignyi*/*felixarabica*.** Parapatric ranges along the Dead Sea Rift in the Levantine region [12-13]. Considered as Pleistocene contacts given the suitable conditions in the area for both species (Fig. 5, [12]). Very limited admixture based on genome-wide data (<5%) and syntopy of mitochondrial haplotypes at a single Israeli locality, where three individuals were suspected as hybrids [13].

Hyla felixarabica

PHYLOGEOGRAPHY IN BRIEF – Mitochondrial and nuclear analyses identified strong genetic differentiation between the geographically disrupted populations of the Levant and the southwestern parts of the Arabian Peninsula [5, 13]. The conditions in the Levant appear to have remained stable for the species since the LGM (Fig. 5, see also [12]).

CONTACT ZONES – ***H. savignyi*/*felixarabica*.** See the account for *H. savignyi*.

Hyla meridionalis

PHYLOGEOGRAPHY IN BRIEF – A genetically-rich species, naturally endemic to N-Africa, and composed of two main Pleistocene clades: one restricted to the Algerian coast, and the second diversified throughout Morocco [17, 22-23]. The European and insular populations from the Canaries and the Balearic archipelago result from introductions of various Moroccan lineages [17]. SDM analyses support suitable LGM conditions along the Mediterranean coast, which could have acted as a large glacial refugium (Fig. 5), as previously suggested from the high genetic diversity of these populations [23].

CONTACT ZONES – **(1) *H. meridionalis*/*carthaginiensis*.** Narrow transition along the E-Algerian coast, considered a Pleistocene contact given that it corresponds to putative refugial ranges for both species according to SDM analyses (Fig. 5) and the high genetic diversity [23]. Based on genome-wide data, admixture was confirmed at a single locality (near Guelma), although a sampling gap remains between the Gulf of Stora and the Gulf of Bougie [23]. **(2) *H. meridionalis*/*molleri*.** See the account for *H. molleri*.

Hyla carthaginiensis

PHYLOGEOGRAPHY IN BRIEF – Restricted to E-Algeria and N-Tunisia, *H. carthaginiensis* features high nuclear and mitochondrial diversity, suggesting moderate effects of the glaciations on population dynamics, and putatively large refugial areas along the Mediterranean coast, as for its sister species *H. meridionalis* [23]. The SDM analyses confirmed high probabilities of occurrence during the LGM along the N-African coast (Fig. 5).

CONTACT ZONES – **(1) *H. meridionalis*/*carthaginiensis*.** See the account for *H. meridionalis*.

References

1. Dufresnes, C. *et al.* Conservation phylogeography: does historical diversity contribute to regional vulnerability in European tree frogs (*Hyla arborea*)? *Mol. Ecol.* **22**, 5669–5684 (2013).

2. Dufresnes, C. *et al.* Sex-chromosome differentiation parallels postglacial range expansion in European tree frogs (*Hyla arborea*). *Evolution* **68**, 3445–3456 (2014).

3. Stöck, M. *et al.* Cryptic diversity among Western Paleartic tree frogs: Postglacial range expansion, range limits, and secondary contacts of three European tree frog lineages (*Hyla arborea* group). *Mol. Phylogenet. Evol.* **65**, 1–9 (2012).

4. Verardi, A, Canestrelli, D., Nascetti, G. Nuclear and mitochondrial patterns of introgression between the parapatric European tree frogs *Hyla arborea* and *H. intermedia. Ann. Zool. Fenn.* **46**, 247–258 (2009).

5. Dufresnes, C. *et al.* Genomic evidence for cryptic speciation in tree frogs from the Apennine Peninsula, with description of *Hyla perrini* sp. nov. *Front. Ecol. Evol.* **6**, 144 (2018).

6. Dubey, S., Lavanchy, G., Thiébaud, J., & Dufresnes, C. Herps without border: a new newt case and a review of transalpine alien introductions in Western Europe. *Amphibia-Reptilia*, **40**, 13–27 (2019).

7. Dufresnes, C., Dubey, S., Ghali, K., Canestrelli, D., & Perrin, N. Introgressive hybridization of threatened European tree frogs (*Hyla arborea*) by introduced *H. intermedia* in Western Switzerland. *Conserv. Genet.* **16**, 1507–1513 (2005).

8. Dufresnes, C. *et al.* Evolutionary melting pots: a biodiversity hotspot shaped by ring diversifications around the Black Sea in the Eastern tree frog (*Hyla orientalis*). *Mol. Ecol.* **25**, 4285–4300 (2016).

9. Dufresnes, C. *et al.* Timeframe of speciation inferred from secondary contact zones in the European tree frog radiation (*Hyla arborea* group). *BMC Evol. Biol.* **15**, 155 (2015).

10. Dufresnes, C. *et al.* Empirical evidence for large X-effects in animals with undifferentiated sex chromosomes. *Sci. Rep.* **6**, 21029 (2016).

11. Gvoždík, V. *et al.* Speciation history and widespread introgression in the European short-call tree frogs (*Hyla arborea* sensu lato, *H. intermedia* and *H. sarda*). *Mol. Phylogenet. Evol.* **83**, 143–155 (2015).

12. Dufresnes, C., Mazepa, G., Jablonski, D., Sadek, R. A., & Litvinchuk, S. N. A river runs through it: tree frogs genomics supports the Dead Sea Rift as a rare phylogeographic break. *Biol. J. Linn. Soc.* **128**, 130–137 (2019).

13. Gvoždík, V., Moravec, J., Klütsch, C., & Kotlík, P. Phylogeography of the Middle Eastern tree frogs (*Hyla*, Hylidae, Amphibia) as inferred from nuclear and mitochondrial DNA variation, with a description of a new species. *Mol. Phylogenet. Evol.* **55**, 1146–1166 (2010).

14. Gül, S., Kutrup, B., Özdemir, N. Patterns of distribution of tree frogs in Turkey based on molecular data. *Amphibia-Reptilia* **33**, 95–103 (2012).

15. Sánchez-Montes, G., Recuero, E., Marcia Barbosa, A., & Martinez-Solano, I. Complementing the Pleistocene biogeography of European amphibians: Testimony from a southern Atlantic species. *J. Biogeogr.* **46**, 568–583 (2019).

16. Barth, A. *et al.* Mitochondrial uniformity in populations of the treefrog *Hyla molleri* across the Iberian Peninsula. *Amphibia-Reptilia* **32**, 557–564 (2011).

17. Recuero, E., Iraola, A., Rubio, X., Mahordom, A., García-París, M. Mitochondrial differentiation and biogeography of *Hyla meridionalis* (Anura: Hylidae): an unsual phylogeographical pattern. *J. Biogeogr.* **34**, 1207–1219 (2007).

18. Oliveira, M. E., Paillette, M., Rosa, H. D., & Crespo, E. G. A natural hybrid between *Hyla arborea* and *Hyla meridionalis* detected by mating calls. *Amphibia-Reptilia* **12**, 15–20 (1991).

19. Drillon, O., Dufresnes, G., Perrin, N., Crochet, P.-A., Dufresnes, C. Reaching the edge of the speciation continuum: hybridization between three sympatric species of tree frogs (*Hyla*). *Biol. J. Linn. Soc.* **126**, 743–750 (2019).

20. Canestrelli, D., Cimmaruta, R., Nascetti, G. Phylogeography and historical demography of the Italian treefrog, *Hyla intermedia*, reveals multiple refugia, population expansions and secondary contacts within peninsular Italy. *Mol. Ecol.* **16**, 4808–4821 (2007).

21. Bisconti R, Canestrelli D, Colangelo P, Nascetti G. 2011. Multiple lines of evidence for demographic and range expansion of a temperate species (*Hyla sarda*) during the last glaciation. Molecular Ecology 20, 5313–5327.

22. Beddek, M. *et al.* Comparative phylogeography of amphibians and reptiles in Algeria suggests common causes for the east-west phylogeographic breaks in the Maghreb. *PLoS ONE* **13**, e0201218 (2018).

23. Dufresnes, C. *et al.* Diversification and speciation in tree frogs from the Maghreb (*Hyla meridionalis* sensu lato), with description of a new African endemic. *Mol. Phylogenet. Evol.* **134**, 291–299.

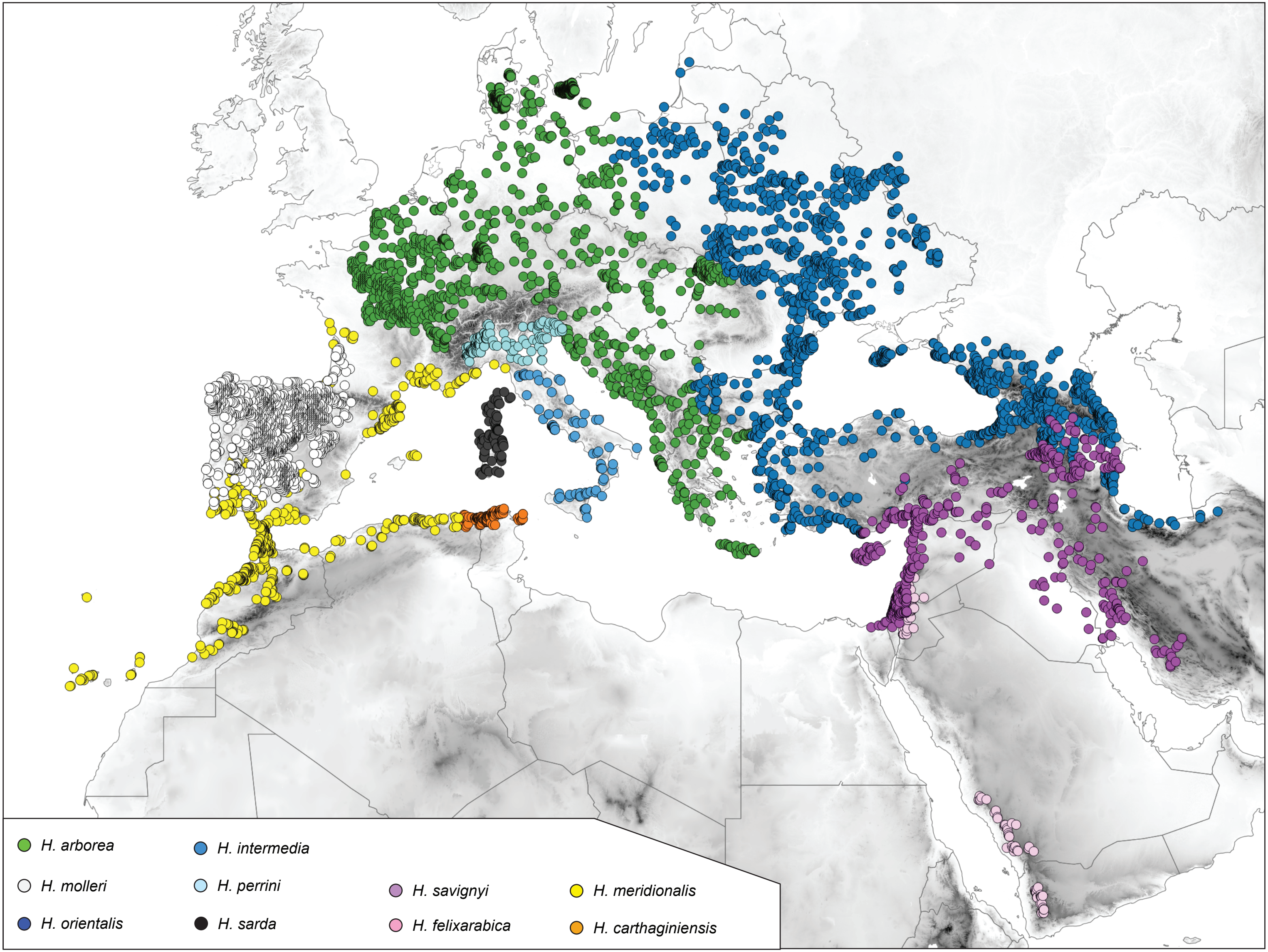

File S3: Occurence records of *Hyla* in the W-Palearctic (excluding confirmed hybrid populations), as used in the SDM analyses. The map was created in QGIS 3.4 (https://qgis.org).

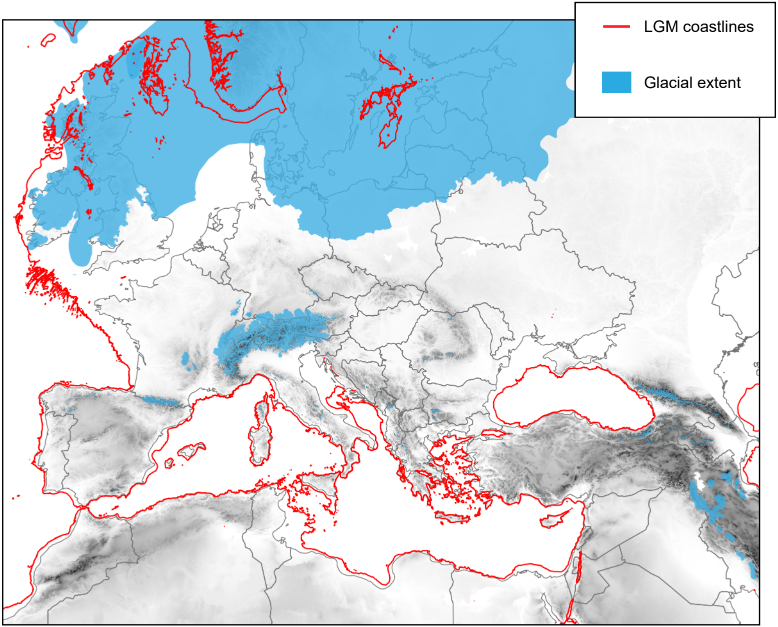

File S4: Glacial extent and coastlines in the W-Palearctic during the Last Glacial Maximum (LGM, 21’000 years ago). The map was created in QGIS 3.4 (https://qgis.org).

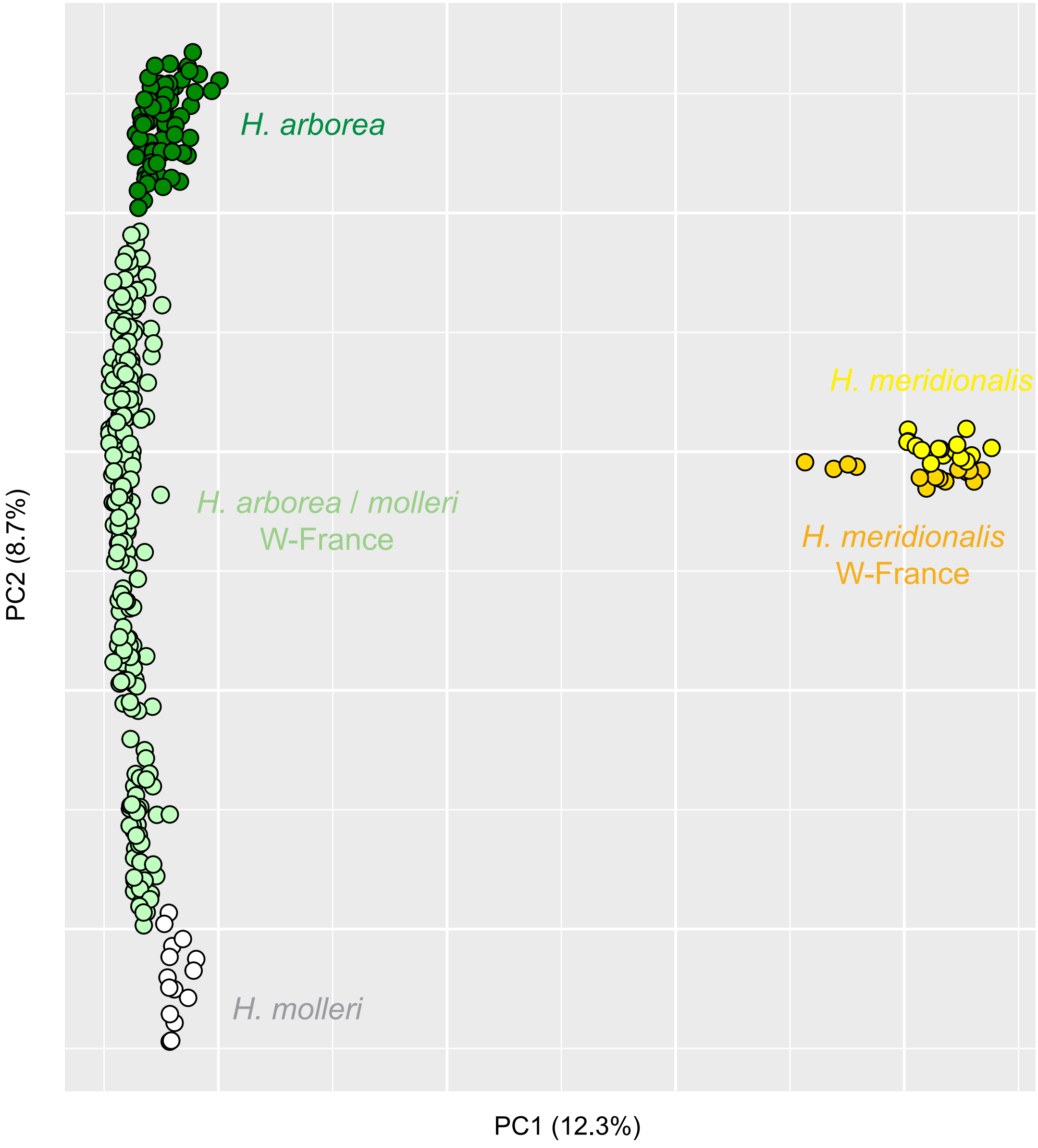

File S5: PCA on individual microsatellite genotypes in tree frogs from W-France and reference populations.

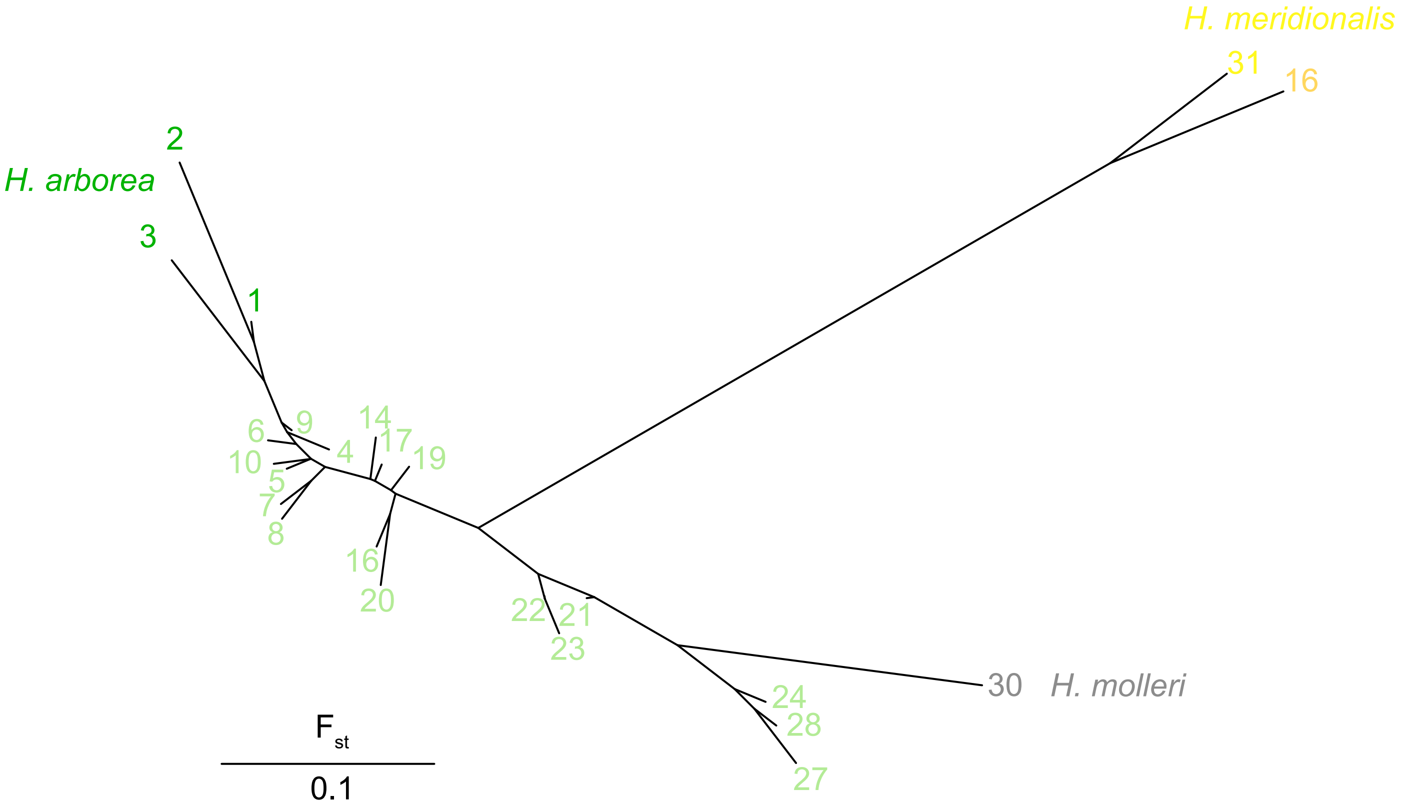

File S6: Neighbor-Joining tree of pairwise genetic distance (F_{st}) between tree frog populations with n ≥ 5. Reference populations (1-3, 30-31) are indicated with the corresponding species names.

File S7: Number of localities, performance metrics for parameter settings in respect to regularization multiplier and feature classes (l: linear, q: quadratic, p: product, t: threshold, h: hinge) of the final models, and relative contribution (%) of each variable in the present time SDMs. *arb*: *H. arborea*; *ori*: *H. orientalis*; *mol*: *H. molleri*; *int*: *H. intermedia*; *per*: *H. perrini*; *sar*: *H. sarda*; *sav*: *H. savignyi*; *fel*: *H. felixarabica*; *mer*: *H. meridionalis*; *car*: *H. carthaginiensis*.

	arb	***car***	***fel***	***int***	***mer***	***mol***	***ori***	***per***	***sar***	***sav***
Number of localities	1862	154	133	78	606	1233	1563	151	74	507
Number of localities after filtering	844	52	82	57	351	744	1086	86	53	330
Regularization multiplier	1.0	3.5	0.5	1.0	2.5	3.5	2.5	6.0	1.0	6.0
Feature classes	t	h	lq	t	qh	t	lqpth	t	qp	h
Partial ROC	0	0	0	0	0	0	0	0	0	0
Omission rate 5%	0.046	0.040	0.073	0.043	0.037	0.050	0.046	0.049	0.042	0.045
AICc	25418	1053	2071	1293	8987	20020	26870	2058	979	9050
Delta AICc	0	0	0	0	0	0	0	0	0	0
Annual mean temperature (Bio1)	–	0	–	–	–	–	–	–	–	4.2
Mean diurnal range (Bio 2)	–	0	0.7	–	–	–	0.9	–	–	0.1
Isothermality (Bio 3)	–	11.4	–	–	0.8	–	–	–	–	0
Temperature seasonality (Bio 4)	58.8	5.7	–	37.1	0.5	–	–	42.8	61.8	2.5
Maximum temperature of warmest month (Bio 5)	–	0.4	33.2	–	–	–	6.6	–	–	–
Minimum temperature of coldest month (Bio 6)	–	0.7	–	–	0.8	–	–	–	–	–
Temperature annual range (Bio 7)	–	–	5.0	–	–	–	42.9	–	–	–
Mean temperature of wettest quarter (Bio 8)	–	0	–	–	0.3	–	0.4	–	–	2.7
Mean temperature of driest quarter (Bio 9)	–	–	0.9	23.2	–	62.2	–	8.8	–	–
Mean temperature of warmest quarter (Bio 10)	–	–	–	–	–	–	–	–	–	–
Mean temperature of coldest quarter (Bio 11)	–	–	3.9	–	–	–	34.9	–	–	–
Annual precipitation (Bio12)	–	–	7.2	–	–	–	–	–	–	46.2
Precipitation of wettest month (Bio 13)	–	–	–	–	–	–	–	–	–	–
Precipitation of driest month (Bio 14)	–	–	2.3	–	–	–	–	–	38.2	–
Precipitation seasonality (Bio 15)	41.2	12.5	0.7	–	34.5	14.5	0.4	–	–	2.8
Precipitation of wettest quarter (Bio 16)	–	–	–	–	–	–	–	–	–	–
Precipitation of driest quarter (Bio 17)	–	–	–	–	0.7	–	1.7	–	–	–
Precipitation of warmest quarter (Bio 18)	–	0.1	2.8	–	–	–	–	–	–	1.1
Precipitation of coldest quarter (Bio 19)	–	–	–	–	37.6	–	0.5	–	–	–
Altitude	–	7.4	4.5	–	5.8	23.3	1.0	25.5	–	5.2
Aridity index	–	59.2	1.2	–	11.0	–	4.1	–	–	3.9
Aspect	–	0.1	0.7	–	0.4	–	0	–	–	0.2
Exposition	–	0	0.6	–	0.1	–	0.1	–	–	0
Habitat homogeneity	–	0	4.1	–	0.8	–	1.8	–	–	17.6
Slope	–	0	0.5	–	0	–	0.4	–	–	0
Terrain roughness index	–	0.1	1.5	–	0.1	–	0.7	–	–	0
Tree coverage percent	–	2.5	30.2	39.7	6.4	–	3.6	22.8	–	13.5
